# Differential Expression of Secreted Phosphoprotein 1 in the Motor Cortex among Primate Species and during Postnatal Development and Functional Recovery

**DOI:** 10.1371/journal.pone.0065701

**Published:** 2013-05-31

**Authors:** Tatsuya Yamamoto, Takao Oishi, Noriyuki Higo, Shigeo Murayama, Akira Sato, Ichiro Takashima, Yoko Sugiyama, Yukio Nishimura, Yumi Murata, Kimika Yoshino-Saito, Tadashi Isa, Toshio Kojima

**Affiliations:** 1 Human Technology Research Institute, National Institute of Advanced Industrial Science and Technology (AIST), Tsukuba, Ibaraki, Japan; 2 Graduate School of Comprehensive Human Science, University of Tsukuba, Tsukuba, Ibaraki, Japan; 3 Research Fellow of the Japan Society for the Promotion of Science, Chiyoda-ku, Tokyo, Japan; 4 Core Research for Evolutional Science and Technology (CREST), Japan Science and Technology Agency, Kawaguchi, Saitama, Japan; 5 Department of Cellular and Molecular Biology, Primate Research Institute, Kyoto University, Inuyama, Aichi, Japan; 6 Precursory Research for Embryonic Science and Technology (PRESTO), Japan Science and Technology Agency, Kawaguchi, Saitama, Japan; 7 Department of Neuropathology, Tokyo Metropolitan Institute of Gerontology, Itabashi-ku, Tokyo, Japan; 8 Computational Systems Biology Research Group, Advanced Science Institute, RIKEN, Yokohama, Kanagawa, Japan; 9 Department of Developmental Physiology, National Institute for Physiological Sciences, Okazaki, Aichi, Japan; 10 Research Equipment Center, Hamamatsu University School of Medicine, Hamamatsu, Shizuoka, Japan; Tokyo Medical and Dental University, Japan

## Abstract

We previously reported that secreted phosphoprotein 1 (SPP1) mRNA is expressed in neurons whose axons form the corticospinal tract (CST) of the rhesus macaque, but not in the corresponding neurons of the marmoset and rat. This suggests that SPP1 expression is involved in the functional or structural specialization of highly developed corticospinal systems in certain primate species. To further examine this hypothesis, we evaluated the expression of SPP1 mRNA in the motor cortex from three viewpoints: species differences, postnatal development, and functional/structural changes of the CST after a lesion of the lateral CST (l-CST) at the mid-cervical level. The density of SPP1-positive neurons in layer V of the primary motor cortex (M1) was much greater in species with highly developed corticospinal systems (i.e., rhesus macaque, capuchin monkey, and humans) than in those with less developed corticospinal systems (i.e., squirrel monkey, marmoset, and rat). SPP1-positive neurons in the macaque monkey M1 increased logarithmically in layer V during postnatal development, following a time course consistent with the increase in conduction velocity of the CST. After an l-CST lesion, SPP1-positive neurons increased in layer V of the ventral premotor cortex, in which compensatory changes in CST function/structure may occur, which positively correlated with the extent of finger dexterity recovery. These results further support the concept that the expression of SPP1 may reflect functional or structural specialization of highly developed corticospinal systems in certain primate species.

## Introduction

Secreted phosphoprotein 1 (SPP1), also known as osteopontin, was originally isolated from bone [1], and has been found in many cell types in other tissues including kidney tubule cells, macrophages, activated T cells, and vascular smooth muscle cells [2]–[7]. It is also known to be involved in glial immune function and tumor progression [8]–[10]. However, there have been few reports on the expression of SPP1 in neurons.

We recently found that SPP1 mRNA was abundantly expressed in the motor related area compared to the prefrontal association area of the rhesus macaque by genome-wide gene expression analysis (Sato et al. BBRC 2007). Consequently, we investigated the expression of SPP1 mRNA in the cerebral cortex of the rhesus macaque more intensely, and found a large number of SPP1 mRNA–positive neurons with intense hybridization signals in layer V of the primary motor area (M1) [11]. Most of the positive neurons in the rhesus macaque M1 were presumed to be corticospinal tract (CST) neurons; however, SPP1 mRNA is not expressed in CST neurons of the rat and marmoset [11]. Both physiological and anatomical differences in the CST exist between primates and rodents, and even between the rhesus macaque and marmoset; such differences are thought to underlie differences in finger dexterity [12], [13]. For these reasons, we have suggested that the expression of SPP1 mRNA in the CST neurons of the rhesus macaque is related to the functional/structural specialization of highly developed corticospinal systems, which underlie higher levels of finger dexterity in certain primate species [11].

To further examine this conjecture, in the present study we evaluated SPP1 expression in the motor cortex from three viewpoints: species differences, postnatal development, and functional/structural changes of the CST after a lesion of the lateral CST (l-CST) at the mid-cervical level. We first compared the density of SPP1-positive neurons in M1 between species with highly developed corticospinal systems (i.e., the rhesus macaque, capuchin monkey, and human) and those with less developed corticospinal systems (i.e., the squirrel monkey, marmoset, and rat). We focused mainly on differences in SPP1 mRNA expression in layer V of M1 among three New World monkeys that show marked differences in their manual dexterity: the marmoset, squirrel monkey, and capuchin monkey [14], [15].

We also investigated the expression of SPP1 mRNA during postnatal development in macaque monkeys. Previous studies have shown that both physiological and anatomical changes occur in the CST during postnatal development of the rhesus macaque: the formation of direct CST connections with motoneurons and the increase in CST conduction velocity parallels the postnatal development of fine finger movements [16], [17]. Therefore, we compared the density of SPP1 mRNA–positive neurons in M1 of macaque monkeys at ages ranging from postnatal day 10 (P10) to P2450 (6.7 y), and examined how the temporal change in SPP1 mRNA expression is related to postnatal development of the CST.

Moreover, we investigated the changes in SPP1 mRNA expression in the motor cortex after a lesion of the CST. Our previous studies showed that functional changes in the motor cortex occur during the recovery of finger dexterity after a unilateral lesion of the l-CST at the mid-cervical level [18], and that structural changes of neuronal projections including the CST may be associated with the observed functional changes [19]. Therefore, we considered that a lesion of the l-CST is also an effective experimental model to investigate the relation between SPP1 mRNA expression and the structure/function of the CST.

## Materials and Methods

### Subjects

The present study consisted of three separate experiments. The animals and human brains used in each experiment are described in Table 1–3. The following analyses were performed. First, species differences were analyzed based on differences in SPP1 expression among six mammalian species: Wistar rats, common marmosets, squirrel monkeys, tufted capuchin monkeys, rhesus macaques, and humans (Table 1). Second, a developmental analysis was conducted based on differences in SPP1 mRNA expression at ages ranging from infancy to adulthood in rhesus and Japanese macaques (Table 2). Third, an l-CST lesion analysis was performed based on differences in SPP1 mRNA expression in rhesus macaques before and after an l-CST lesion at the mid-cervical level (Table 3).

**Table 1:** Subjects used in species differences analysis.

Species	Number	Weight	Sex (male ∶ female)
Wistar rat (*Rattus norvegicus*)	4	260–380 g	3∶1
Common marmoset (*Callithrix jacchus*)	3	300–360 g	2∶1
Squirrel monkey (*Saimiri sciureus*)	3	620–850 g	2∶1
Tufted capuchin monkey (*Cebus apella*)	2	1.9–4.0 kg	1∶1
Rhesus macaque monkey (*Macaca mulatta*)	3	3.0–8.5 kg	2∶1
Human (*Homo sapiens*; tissue samples)	5	76, 79, 92, 71, and 76 year old	Male
		with no neurological findings	

**Table 2:** Subjects used in developmental analysis.

Species	Age	Weight	Sex
Rhesus macaque monkey (*Macaca mulatta*)	10, 31, 70, 90, 183, 365, 365,	0.75–8.5 kg	8∶4
	547, 893, 1241, 2233, 2450 day		
Japanese macaque monkey (*Macaca fascata*)	8, 70, 184, 229, 365, 730 day	0.56–4.5 kg	Male

**Table 3:** Subjects used in l-CST lesion analysis using adult rhesus macaque monkeys.

Condition	Number	Weight	Sex
Intact	3	3.0–8.5 kg	2∶1
Lesion	9	2.7–4.2 kg	6∶3

Wistar rats were purchased from a local provider (Japan SLC, Inc. Hamamatsu,Japan). Eleven of the 21 rhesus macaques were also purchased from a local provider (Hamri Co., Ltd., Ibaraki, Japan, and Japan Wild Animal Research Center, Kagoshima, Japan). Other rhesus macaques were bred at the Primate Research Institute of Kyoto University (n = 9) and Nihon University School of Medicine (n = 1), Tokyo, Japan. Marmosets were bred at RIKEN Center for Molecular Imaging Science, Kobe, Japan (n = 1), and the Primate Research Institute of Kyoto University, Inuyama, Japan (n = 2). Capuchin monkeys and squirrel monkeys were bred at the Primate Research Institute of Kyoto University. Japanese macaques were bred at the Primate Research Institute of Kyoto University (n = 5) and Juntendo University School of Medicine, Tokyo, Japan (n = 1). Human brain and spinal cord tissues were provided by the Brain Bank for Aging Research Project at the Tokyo Metropolitan Institute of Gerontology, Tokyo, Japan.

All animal experiments were approved by the Animal Care and Use Committee of the Primate Research Institute of Kyoto University, the National Institute of Advanced Industrial Science and Technology, and the National Institutes of Natural Sciences, Japan. These guidelines are based on the recommendations of the National Research Council as published in the ILAR “Guide for the Care and Use of Laboratory Animals”, and all research procedures followed the recommendations of the ILAR Guide, therefore also consistent with the recommendations of the Weatherall Report on “The Use of Non-Human Primates in Research”. Adequate measures were taken to minimize pain or discomfort in accordance with these guidelines. The monkeys used in the species difference and developmental analyses were group-housed. They were housed together with their birth mothers until weaning at the age of about one year. The rhesus macaques used in the l-CST lesion study were housed in adjoining individual primate cages (600×750 mm in area and 900 mm in height) allowing social interactions, under controlled conditions of humidity, temperature and light, and they were monitored daily by the researchers and the animal care staff to check the conditions of health and welfare. Environmental enrichment consisted of commercial toys. A commercial primate diet and fresh fruit/vegetable were provided daily, and water was provided in a drinking bottle and freshened daily. Endpoint criteria, as defined by the study protocol, were used to determine when animals should be humanely euthanized.

Human tissue experiments were approved by the Institutional Review Board for using Human Derivatives for Biomedical Research of the National Institute of the AIST and the Ethical Committee of the Tokyo Metropolitan Institute of Gerontology. Written informed consents were obtained for all human tissue samples, and all the samples were anonymized and cannot be traced back to individual patients.

### L-CST lesion analysis: Surgery and behavioral tests

Prior to the l-CST lesion analysis, a lesion was made around the dorsolateral funiculus where most of the corticospinal tract fibers descend, as previously described [18], [20]. Briefly, the animals were anesthetized with ketamine hydrochloride (10 mg/kg, i.m.) and xylazine (1 mg/kg, i.m.), and then maintained with sodium pentobarbital (20 mg/kg, i.v.). The border between cervical segments 4 (C4) and C5 was exposed by laminectomy of the C3 and C4 vertebrae, and a transverse opening was made in the dura. The lesion was made under a surgical microscope as follows. A small opening in the pia mater was made at the lateral convexity of the spinal cord. A horizontal cut in the mediolateral direction relative to the lateral funiculus was made by inserting a minute L-shaped hook with a maximum possible insertion depth of 5 mm, which corresponded to the distance from the lateral convexity of the spinal cord to the midline. Then, using a watchmaker's forceps, the dorsal part of the lateral funiculus was transected from the dorsal root entry zone ventral to the level of the horizontal strip. Using the forceps, the lesion was extended ventrally to the most lateral part of the lateral funiculus.

The extent of lesion in each monkey was quantified as described previously [19]. The C4 and C5 segments were cut into 50 µm sections, and Klüver-Barrera staining was performed to visualize the lesion. We measured the area of the intact part of the lateral and ventral funiculus on the lesioned side (α) and the whole area of the lateral and ventral funiculus on the intact side (β). Then we calculated 100×(1−α/β), which yielded the percentage of the lesion extent. The average lesion extent percentage in all monkeys used in the present study was 59.67±21.06% (mean ± standard deviation [SD]). This value is consistent with those of monkeys we used in our previous study, in which an anatomical tracer experiment confirmed that most of the l-CST fibers were interrupted [18].

All monkeys exhibited limb paralysis after the lesion, but motor recovery to the level of intact monkeys was observed in a retrieval task in which the monkeys had to reach, grasp, and retrieve a small cubical piece of sweet potato (about 7 mm ×7 mm ×7 mm) through a narrow vertical slit by using the index finger and thumb during the first month after the lesion [20]. Thus, we defined 2 weeks (n = 4 monkeys) to 1 month (n = 1 monkey) after the lesion as the early recovery stage and 3 months (n = 4 monkeys) after the lesion as the late recovery stage, as in our previous reports [18], [19]. In this study, to assess rigorously the extent of finger dexterity after the lesion, a success trial was defined as any trial that resulted in the removal of the food from the pin using the precision grip between the tip of the index finger and thumb, without dropping the food.

### Tissue preparation

#### Experimental animal tissues

All animals were deeply anesthetized with sodium pentobarbital (Nembutal; 35–50 mg/kg for rhesus macaques, tufted capuchins, squirrel monkeys, and marmosets, 65 mg/kg for rats, i.v.). Then the animals were perfused through the ascending aorta with 0.5 L of ice-cold saline containing sodium heparin (1000 units/mL), followed by ice-cold fixative consisting of 4% paraformaldehyde (PFA) and 0.1% glutaraldehyde in phosphate buffer (PB), pH 7.4 (volumes were 3–4 L for rhesus macaques, 2–3 L for tufted capuchins, 600–900 mL for squirrel monkeys, and 300 mL for marmosets and rats). During perfusion, the animals' heads were chilled with crushed ice. After perfusion, the brains were immediately removed and dissected into 5 mm tissue blocks and then immersed in a post-fixative solution containing 2% PFA and 5% sucrose in PB for several hours, followed by successive immersions in 10%, 20%, and 30% sucrose in PB. The brain blocks were mounted in optical cutting temperature (OCT) compound (Miles Inc., Elkhart, IN, USA), rapidly frozen in a dry ice/acetone bath, and stored at −80°C until dissection.

#### Human tissue

Within 48 h after death, human brains were removed and immersed in a fixative solution containing 4% PFA for 12 h. Then they were blocked and immersed in a post-fixative solution containing 2% PFA and 5% sucrose in PB for several hours, followed by successive immersions in 10%, 20%, and 30% sucrose in PB. The brain blocks were frozen as described above.

### Section preparation

#### Cerebral cortex

Coronal sections (16 µm thick) were cut from the forelimb/hand area of animal and human M1 using a cryostat (CRYOCUT 3000; Leica, Nussloch, Germany). Anatomical atlases and original research studies were used to guide the preparation of sections from the rat [21], [22], marmoset [23], [24], and squirrel monkeys [25], [26], based on brain structure and cytoarchitecture visualized by Nissl staining. Similarly, sections from the rhesus macaque [27], [28], capuchin monkey [29], and human [30] were prepared from tissue surrounding the central sulcus. Sections of premotor cortex (PM) forelimb region from the rhesus macaque were prepared from tissue surrounding spur of the arcuate sulcus [27], [31].

#### Subcortical structures

Coronal sections were prepared from the red nucleus of the brainstem and transverse sections were prepared from the cervical spinal cord of the rat [21], [32], marmoset [23], squirrel monkey [26], capuchin monkey [14], [33], rhesus macaque [27], pig [34], [35], and human [36] based on current literature or anatomical atlases. We prepared sections from C8 of the spinal cord in all animals except for humans, for which we used C6 because the ventral horn of the human C8 is susceptible to age-related degeneration.

### *In situ* hybridization histochemistry

*In situ* hybridization (ISH) was performed as described previously [11], [37]. SPP1-specific RNA probes for each of five species (capuchin monkey, human, rhesus macaque, marmoset, and rat) were prepared by reverse transcription-polymerase chain reaction (RT-PCR) and conventional TA cloning techniques. The primer sequences were atgatggccgaggtgatagtgt and aagatgcactatctaattcatg for capuchin monkey SPP1 (cDNA from capuchin monkey), and tgaatctgatgaactggtcactg and accagcatatcttcatggctgt for human SPP1 (targeted to positions 483–959 in AK290104; cDNA from human). The probe sizes were 465 and 477 bp for the capuchin monkey and human, respectively. The primer sequences and probe sizes for the rhesus macaque, marmoset, and rat were described previously [11]. Probes made from the capuchin monkey SPP1 sequence were also used for ISH in the squirrel monkey, which is a New World monkey phylogenetically closely related to the capuchin monkey [38], [39]. For the squirrel monkey, we also used probes made from the SPP1 sequence of the marmoset, another New World monkey [38], [39]. No differences were observed between the results obtained using the capuchin monkey and marmoset probes; therefore, the combined data from both probes were used for quantification. For the developmental analysis, probes made from the SPP1 sequence of the rhesus macaque were also used for the phylogenetically closely related Japanese macaque [38], [39], as in our previous report [40]. The results from both species were consistent in each postnatal stage. Control sections for all probes showed no specific staining, except in the case described below. In addition, double-labeling using ISH for SPP1 mRNA and immunofluorescence with a mouse anti-SMI 32 monoclonal antibody (SMI-32P, Covance, Emeryville, CA, USA), a marker antibody of medium- and heavy-chain nonphosphorylated neurofilaments present in the subcortically projecting layer V neurons, a subset of which are corticospinal motor neurons [41], [42], was performed as described previously [37], except that ISH preceded immunofluorescence labeling.

### Immunohistochemistry

In one of five human tissues, prominent hybridization signals for SPP1 mRNA were observed in layer V of the M1 (data not shown), similarly to the result in rhesus macaque M1. To perform a population analysis of SPP1-positive neurons in human M1, we used immunohistochemistry (IHC) instead of ISH because some of our samples were unsuitable for ISH owing to mRNA degradation during tissue preparation. To confirm that the SPP1 protein expression pattern was equivalent to that of SPP1 mRNA, we performed IHC in the rhesus macaque (n = 3). IHC was performed with a mouse anti-SPP1 monoclonal antibody (sc-21742, Santa Cruz Biotechnology, Inc., Santa Cruz, CA, USA) by using the Vectastain Elite ABC Mouse IgG Kit (PK-6102, Vector Laboratories, Inc., Burlingame, CA, USA) according to the manufacturer's instructions. In addition, double-labeling IHC was performed in human M1, in which enzyme labeling for SPP1 protein preceded immunofluorescence labeling for SMI 32. The SMI 32 primary antibody, which was conjugated to Alexa Fluor 488 by using the Zenon mouse IgG labeling kit (Invitrogen, Carlsbad, CA, USA), was used according to the manufacturer's instructions.

#### Antibody characterization

The mouse monoclonal antibody to SPP1 (clone no.: AKm2A1), prepared against mouse recombinant SPP1, reacts strongly with a single protein band at about 70 kDa on Western blot (manufacturer's technical information). Moreover, the distribution of SPP1 protein expression revealed by this antibody was identical to that of SPP1 mRNA expression. We confirmed secondary antibody specificity by using coronal sections incubated without primary antibody; no specific signal was observed. In another control experiment, primary antibodies were preabsorbed with SPP1 protein (O2260, Sigma, St. Louis, MO, USA) for 12 h at 4°C; staining was considerably weaker than that seen with the normal primary antibody.

The mouse monoclonal antibody to SMI 32 recognizes nonphosphorylated epitopes on medium (170 kDa) and heavy (200 kDa) molecular weight subunits of neurofilament H in immunoblots of the mammalian brainstem and spinal cord tissue [43], [44]. This antibody is used to visualize neuronal cell bodies, dendrites, and some thick axons in the central and peripheral nervous systems, although it does not reveal thin axons (manufacturer's information). In addition, this antibody primarily labels the cell bodies and dendrites of a subset of pyramidal neurons with distant axonal projections, such as the corticocortical neurons in layer III and a subset of subcortically projecting neurons in layer V of the monkey and human cortex [41], [42], [45]. In agreement with these previous studies, the antibody showed the same cellular morphology and distribution pattern in our M1 analysis. We confirmed secondary antibody specificity by using coronal sections incubated without primary antibody; no specific signal was observed (data not shown).

### Image acquisition

Images of ISH and IHC sections were acquired with an Olympus BX60 microscope equipped with a 3CCD color video camera (DXC-950; Sony, Tokyo, Japan) and digitized with an image analysis system (MCID; Imaging Research, St. Catherines, Ontario, Canada). Image editing, which was accomplished in Adobe Photoshop CS3 (Adobe Systems Inc., San Jose, CA, USA), involved cropping, resizing, adjusting brightness and contrast, and removing obvious contaminations.

### Quantification

In analyzing species differences, the number of SPP1-positive neurons was counted automatically by MCID in a 2-mm-wide column that sampled each M1 layer (II, III, V, and VI). The columns were chosen from cortical areas without nonspecific and patchy staining. The ratio of the optical density (OD) of neurons to the OD of the background staining in the subcortical white matter was calculated as a percentage. Neurons that showed an OD greater than 150% of the OD of background staining and were also larger than 100 µm^2^ in area were counted. The raw counts of SPP1-positive neurons were corrected for double counting by the method of Abercrombie [46]. Three columns were measured in each section, and two sections were measured in each subject. In the squirrel monkeys, two sections that showed a strong signal when hybridized with the capuchin monkey sense probe were excluded from the analysis; we used 10 sections hybridized with either the capuchin monkey (n = 4 sections) or marmoset probes (n = 6 sections). Because we obtained the human data from sections stained by IHC instead of ISH, and used different protocols to prepare the animal and human tissue, we plotted the human data separately from the data obtained from other species.

Moreover, in the analysis of species differences, we examined the relationship between the cell body size and signal intensity of SPP1-positive neurons. Only neurons containing a nucleus were measured in laminae III and V of both the capuchin monkey and human M1 by manually tracing cell boundaries in microscopic images.

The number of SPP1-positive neurons in both the developmental and post l-CST lesion analyses was determined by sampling from all layers of the cortex with MCID; most of the positive neurons were located in layer V. We also investigated the relationship between the cell body size of Nissl-stained and SPP1-positive neurons in both the developmental and l-CST lesion analyses. This was performed to determine whether SPP1 mRNA was expressed in a subpopulation of neurons with large cell bodies among all layer V neurons in M1 of each aged monkey in the developmental study and in the ventral premotor cortex (PMv) of each monkey in the l-CST lesion study. To calculate the average size of the largest Nissl-stained neurons, we used the same number of neurons as there were SPP1-positive neurons in each monkey (e.g., if 10 SPP1-positive neurons were observed in a measurement field, we used the 10 largest Nissl-stained neurons for analysis). In microscopic images, we manually traced the cell boundaries of SPP1-positive and Nissl-stained neurons containing a nucleus in three 900×680 µm rectangles. Among all measurements in the developmental and post l-CST lesion analyses, a maximum of 35 SPP1-positive neurons showed an OD greater than 150% of that of the background staining in the pericellular matrix and were larger than 100 µm^2^ in area. To obtain sufficient data for the analysis, we visually selected 45 Nissl-stained neurons with relatively large cell bodies and then manually traced their cell boundaries. Data from monkeys without SPP1 expression were not used in this analysis. The slope difference test in both the developmental and post l-CST lesion analyses was performed using Microsoft Excel 2007 (Microsoft, Redmond, WA, USA); other statistical analyses were performed using GraphPad Prism v 5.01 (GraphPad Software, San Diego, USA).

## Results

### Differential expression of SPP1 among species in layer V of M1

**Figure 1:**
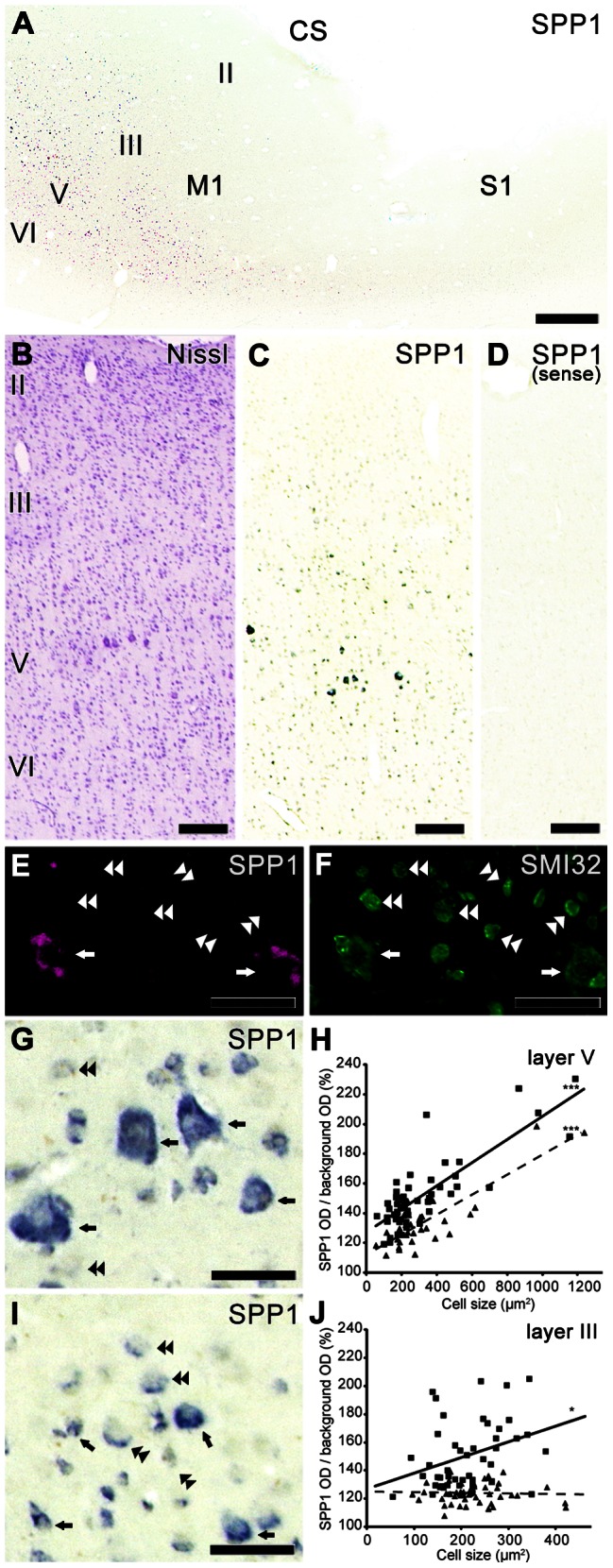
SPP1 mRNA expression in the sensorimotor cortex of the capuchin monkey. A: A photograph showing SPP1 mRNA expression around the central sulcus (CS) in the capuchin monkey. B, C: Nissl-stained (B) and adjacent sections in the primary motor cortex (M1) of the capuchin monkey showing the distribution of SPP1 mRNA-positive neurons (C). D: A control section hybridized with the SPP1 mRNA sense probe. E, F: Double-labeling study with SMI32, an antibody against non-phosphorylated neurofilament H. E: Localization of SPP1 mRNA-positive neurons in layer V of the capuchin monkey M1. F: SMI 32 immunoreactivity in the same section as (E). Arrows indicate SPP1-mRNA-positive neurons showing SMI 32 immunoreactivity. Double arrowheads indicate SMI 32 immunoreactive neurons with no SPP1-mRNA expression. G-J: High-magnification photomicrographs of SPP1 mRNA-positive neurons and scattergram showing the relationship between SPP1 mRNA signal intensity and size of the neuronal cell bodies in layers V (G, H) and III (I, J). Arrows and double arrowheads in (G) and (I) indicate neurons showing intense and weak signals, respectively. In (H) and (J), the number of neurons examined was 57 and 34 in layer V, and 46 and 50 in layer III. *P<0.05, ***P<0.0001, according to linear regression analysis. Squares and triangles are data points from each capuchin monkey. The solid line and dashed line are linear approximations of the data represented by the squares and triangles, respectively. S1, primary somatosensory cortex. II–VI, layers II–VI of the cerebral cortex. Scale bars  = 1 mm in A; 200 µm in B–D; 50 µm in E–G, I.

We first investigated whether SPP1 mRNA was expressed in the CST neurons of the capuchin monkey, a dexterous New World monkey, as we reported in the rhesus macaque, a dexterous Old World monkey [11]. Similar to the results in the rhesus macaque, prominent SPP1 mRNA hybridization signals were mainly observed in layer V of capuchin M1, and weaker hybridization signals were observed in layers III and VI (Fig. 1A–C, Table 4). Signals in the adjacent primary somatosensory cortex (S1) were substantially weaker than those in M1 (Fig. 1A). Control sections showed no specific staining (Fig. 1D). We then performed a double-labeling immunofluorescence experiment with SMI 32, a specific marker for the subcortically projecting neuron subpopulation in layer V, a subset of which are CST neurons [41], [42]. Virtually all SPP1 mRNA–positive neurons were labeled with SMI 32 in layer V of the capuchin monkey M1 (arrows in Fig. 1E, F), but not vice versa (double arrowheads in Fig. 1E, F). Moreover, high-magnification photomicrographs showed that intense hybridization signals were frequently observed in large neurons in layer V of the capuchin monkey M1 (arrows in Fig. 1G), whereas weak hybridization signals were mainly observed in small neurons (double arrowheads in Fig. 1G). A scattergram analysis indicated a significant positive correlation between the intensity of SPP1 mRNA expression and neuron size (Fig. 1H). Because the neuronal population with the largest cell bodies in layer V of M1 consists of CST neurons [47], [48], these results suggest that SPP1 is preferentially expressed in CST neurons of the capuchin monkey, consistent with our recent observations in the rhesus macaque [11]. In the rhesus macaque, the regions positive for SPP1 protein were identical to those showing SPP1 mRNA expression (Table 4).

**Table 4:** Summary of secreted phosphoprotein 1 (SPP1) expression in the six species examined.

	Rat (mRNA)	Common marmoset (mRNA)	Squirrel monkey (mRNA)	Capuchin monkey (mRNA)	Rhesus macaque monkey (mRNA)	Rhesus macaque monkey (protein)	Human (protein)
Primary motor cortex (forelimb area: layer V)	−	−	+	++	+++	+++	+++
Brainstem (red nucleus)	+++	+++	+++	+++	+++	+++	+
Spinal cord (cervical cord)	++	++	++	++	++	++	++

Signal intensity was graded by visual evaluation as follows: +++, intense; ++, moderate; +, weak; −, not detectable. The SPP1 mRNA expression pattern data in the rat, marmoset, and rhesus macaque are from our previous report [11]

**Figure 2:**
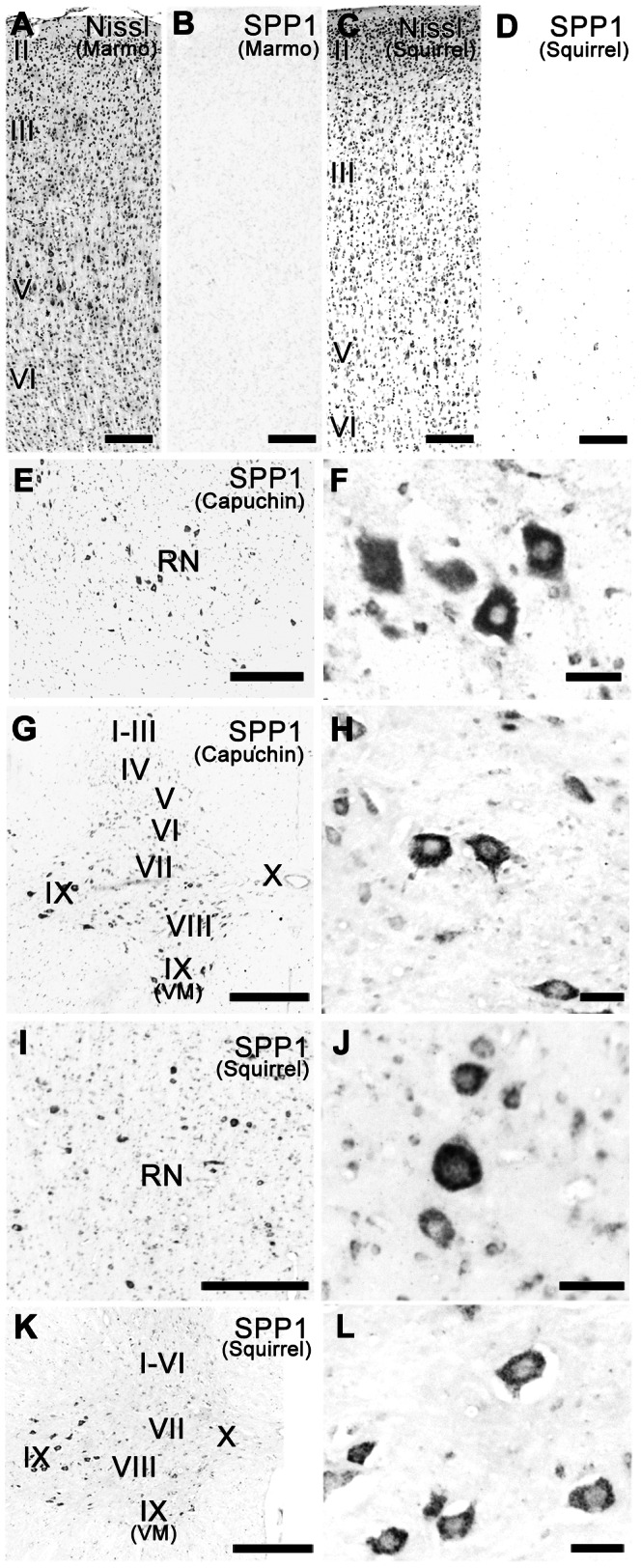
SPP1 expression in the motor cortex of the marmoset and squirrel monkey, and the brainstem and spinal cord of New World monkeys. A, B: Nissl-stained (A) and adjacent sections of the marmoset primary motor cortex (M1) hybridized with the SPP1 antisense probe (B). C, D: Nissl-stained (C) and adjacent sections of the squirrel monkey M1 hybridized with the SPP1 antisense probe (D). E, F: Low- (E) and high- (F) magnification photomicrographs of SPP1 mRNA-positive neurons in the red nucleus of the capuchin monkey. G, H: Low- (G) and high- (H) magnification photomicrographs of SPP1 mRNA-positive neurons in the eighth cervical spinal segment of the capuchin monkey. I, J: Low- (I) and high- (J) magnification photomicrographs of SPP1 mRNA-positive neurons in the red nucleus of the squirrel monkey. K, L: Low- (K) and high- (L) magnification photomicrographs of SPP1 mRNA-positive neurons in the eighth cervical spinal segment of the squirrel monkey. RN, red nucleus. II–VI, layers II–VI of M1. I–X, layers I–X of the spinal cord. VM, ventral medial nucleus. Capuchin, capuchin monkey. Marmo, marmoset. Squirrel, squirrel monkey. Scale bars = 200 µm in A–D; 500 µm in E, G, I, K; 50 µm in F, H, J, L.

**Figure 3:**
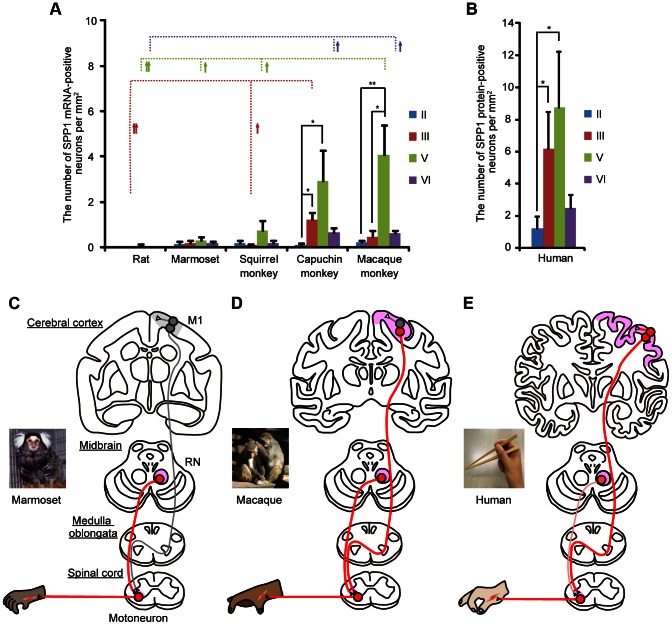
Summary of the results from the species differences analysis. A: The density of SPP1 mRNA-positive neurons in each layer of the primary motor cortex (M1) in five animals: rat, marmoset, squirrel monkey, capuchin monkey, and rhesus macaque (the number of sections in each species was 6, 6, 10, 4, and 6, respectively). The mean density of SPP1 mRNA-positive neurons (± SE) is shown. *P<0.05, **P<0.01, according to a Friedman's one-way analysis of variance (ANOVA) with Dunn's post-hoc tests. †P<0.05, ††P<0.01, according to a Kruskal–Wallis one-way ANOVA with Dunn's post-hoc tests. B: The density of SPP1 protein-positive neurons in each layer of M1 in human (n = 6 sections). The mean density of SPP1 protein-positive neurons (± SE) is shown. *P<0.05, **P<0.01, according to a Friedman's one-way analysis of variance (ANOVA) with Dunn's post-hoc tests. C–E: Schematic diagram of the interspecies differences in SPP1 expression in the projection neurons of motor-related structures including M1, red nucleus (RN), and lower cervical spinal segments. The motor systems are organized in a functional hierarchy from bottom to top: the spinal cord, brainstem, and motor cortex, each associated with increasing levels of complexity. SPP1 was commonly expressed in motor-related areas among all species examined; however, the level of motor hierarchy in which this gene was expressed varied among species. The regions with intense or moderate SPP1 signals are shown as red-filled circles, and those with weak or no signals are shown as gray-filled circles. See Table 4 for expression data details from the RN and spinal cord of each species.

In contrast to expression in the capuchin monkey, almost no SPP1 expression was observed in M1 of the marmoset, a non-dexterous New World monkey (Fig. 2A, B). Similarly, SPP1-positive neurons were rarely counted in the rat M1, as reported previously [49], although both the marmoset and rat M1 contained CST neurons [24], [50]. In M1 of the squirrel monkey, another New World monkey, a small number of large pyramidal neurons (20–30 µm in diameter) with weak SPP1 mRNA hybridization signals were observed (Fig. 2C, D). A quantitative analysis showed that in the squirrel monkey, the density of SPP1-positive neurons in layer V was much smaller than in the capuchin or rhesus macaque but larger than in the marmoset (Fig. 3A). Thus, differential SPP1 expression in M1 was observed among the capuchin monkey, squirrel monkey, and marmoset, even though they are closely related species of New World monkeys. The result in M1 was contrast to that in the red nucleus and spinal cord, in which SPP1 was commonly expressed in these species (Fig. 2E–L; Table 4; our previous report [11]).

**Figure 4:**
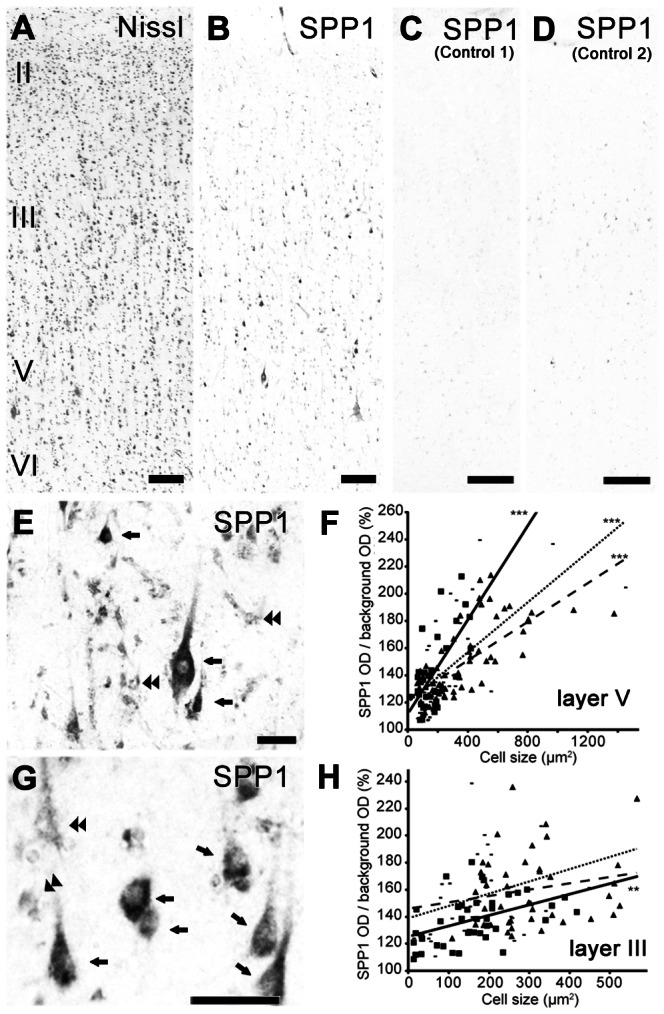
SPP1 expression in the human motor cortex. A, B: Nissl-stained (A) and adjacent sections of the human M1 showing the distribution of SPP1protein-positive neurons (B). C, D: Control section incubated without primary antibody for the SPP1 protein (C) and incubated with primary antibody preabsorbed with SPP1 protein (D). E–H: High-magnification photomicrographs of SPP1 protein-positive neurons and scattergram showing the relationship between SPP1 signal intensity and size of the neuronal cell bodies in layers V (E, F) and III (G, H). Arrows and double arrowheads in (E) and (G) indicate neurons showing intense and weak signals, respectively. In (F) and (H), the number of neurons examined was 33, 56, and 42 in layer V, and 41, 41, and 31 in layer III; **P<0.01, ***P<0.0001, according to linear regression analysis. Squares, triangles, and small rectangles in (F) and (H) are data points from each human tissue sample. The solid, dashed, and dotted lines are linear approximations of the data represented by the squares, triangles, and small rectangles, respectively. Scale bars  = 200 µm in A–D; 50 µm in E, G

In the human M1, prominent SPP1-immunoreactive signals were observed in layer V pyramidal neurons (Figs. 3B and 4A, B, E) relative to controls (Fig. 4C, D). An SMI 32 double-labeling experiment (data not shown) and a quantitative analysis of the correlation between the intensity of SPP1 expression and neuron size (Fig. 4F) in humans showed a result similar to that of capuchin monkeys, suggesting that SPP1 is also preferentially expressed in human CST neurons.

### M1 layer III SPP1 expression in the capuchin monkey and human

In both the capuchin monkey and human M1, quantitative analysis showed that the density of SPP1-positive neurons was significantly higher not only in layer V but also in layer III than in layer II (Fig. 3A, B). This expression pattern was different from that of the rhesus macaque M1, in which little expression was observed in layer III [11]. Thus, we also investigated the morphological features of SPP1-positive neurons in layer III of the capuchin monkey and human M1. The SPP1 signals were mainly observed in the pyramidal neurons of deep layer III (Figs. 1C and 4B). Higher-magnification photomicrographs showed that, unlike layer V, intense (arrows in Figs. 1I, 4G) or weak (double arrow heads in Figs. 1I, 4G) hybridization signals were observed in both large and small-sized neurons of layer III. Moreover, a scattergram analysis of the signals indicated that the correlation coefficients in layer III neurons (capuchin monkey, R^2^ = 0.11, 0.0013; human, R^2^ = 0.093, 0.20, 0.037) were much lower than those in layer V (capuchin monkey, R^2^ = 0.67, 0.72; human, R^2^ = 0.44, 0.43, 0.48) (Figs. 1H, J, and 4F, H). These results indicate that SPP1 expression in layer III neurons correlated mainly with factors other than neuron size, in contrast to the results for layer V neurons.

### SPP1 mRNA expression in the developing macaque monkey M1

**Figure 5:**
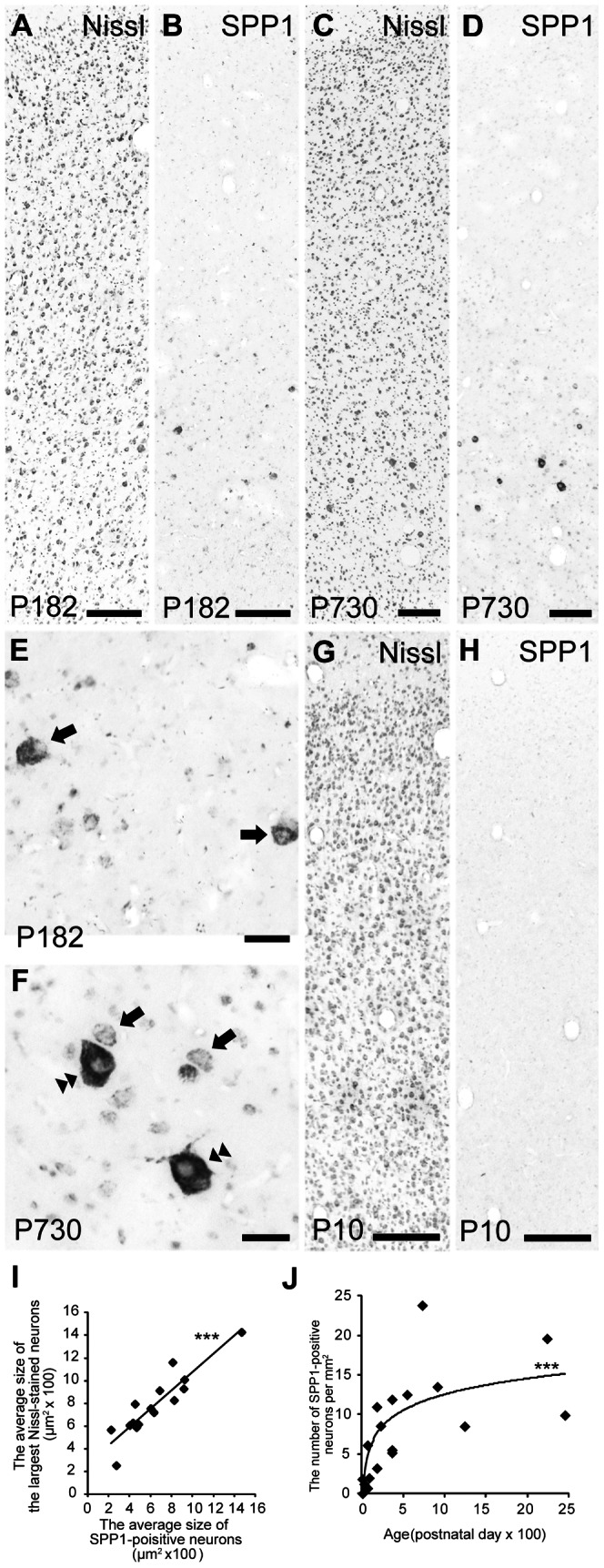
SPP1 expression in the developing macaque monkey. A–D: Nissl-stained and adjacent sections showing the distribution of SPP1 mRNA-positive neurons in the primary motor cortex (M1) of macaque monkey at postnatal day 182 (P182; A, B) and P730 (C, D). E, F: High-magnification photomicrographs of SPP1 mRNA-positive neurons in layer V pyramidal neurons of the macaque monkey M1 at P182 (E) and P730 (F). Arrows and double arrowheads indicate neurons with the cell body size mainly showing intense signals at P182 and P730, respectively. G, H: Nissl-stained and adjacent sections hybridized with the SPP1 antisense probe in the macaque monkey M1 at P10. I: Scattergram showing the relationship between the average size of Nissl-stained neurons with the largest cell bodies and that of SPP1-positive neurons in the macaque monkey at each developmental stage. ***P<0.0001, according to linear regression analysis. J: Scattergram showing the logarithmic correlation between age and the density of SPP1 mRNA-positive neurons in the developing macaque monkey M1. ***P<0.0001, according to Spearman's coefficient of rank correlation. Diamonds represent data points from each monkey. II–VI, layers II–VI of M1. Scale bars  = 200 µm in A–D, G, H; 50 µm in E, F.

During macaque monkey development, prominent hybridization signals for SPP1 mRNA were observed in M1 layer V neurons at several developmental stages, including both P182 (0.5 y) and P730 (2 y; Fig. 5A–F). Although a signal was often observed in medium-sized neurons (10–20 µm in diameter) at P182, many neurons with this cell body size had weak or no signals at P730 (arrows in Fig. 5E, F). At this developmental stage (P730), prominent signals were mainly observed in larger neurons (20–40 µm in diameter; double arrowheads in Fig. 5F). Given that the average size of M1 neuronal cell bodies increases during postnatal development [51], these results indicate that SPP1 is expressed in a subset of M1 neurons with relatively large cell bodies among those at each postnatal developmental stage. This idea was confirmed by results from a scattergram analysis showing a significant positive correlation between the average size of the Nissl-stained neurons with the largest-sized cell bodies (y) and that of SPP1-positive neurons (x) during development (y = 0.81x +272, R^2^ = 0.83, n = 15 monkeys, Fig. 5I); the slope of this regression line did not significantly differ from 1 (P>0.05). Thus, at each postnatal developmental stage, SPP1 is expressed in a subset of M1 neurons with the largest cell bodies, of which the majority may be CST neurons [47], [48].

No expression was observed in M1 at P10 (Fig. 5G, H), even though CST neurons already exist in the macaque monkey M1 at birth [17]. Another scattergram analysis indicated that the density of SPP1 mRNA-positive neurons gradually increased over a long period of time after birth, and it was significantly logarithmically correlated with age (Fig. 5J). The fitted logarithmic function had a time constant of 319.2 days. This equation predicted that SPP1 expression should reach a value within the adult range by 957.6 days (2.6 y; three time constants), which is similar to the reported age (3 y) when CST conduction velocity reaches its adult level [16]. Thus, these results suggest that SPP1 expression in macaque monkey M1 contributes to functional maturation (e.g., conduction velocity elevation) of CST.

### Increased SPP1 expression in PMv of the macaque after an l-CST lesion

**Figure 6:**
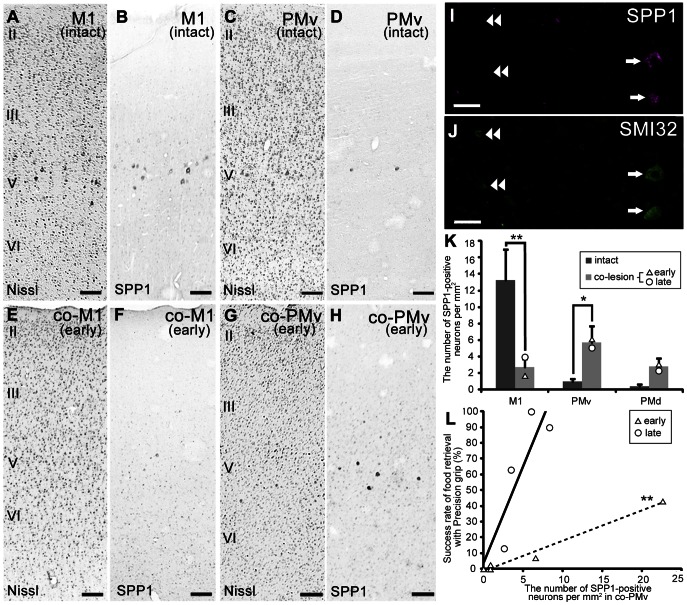
Changes of SPP1 expression after lesion of the lateral corticospinal tract. A–H: Nissl-stained sections and adjacent sections showing the distribution of SPP1 mRNA-positive neurons in the primary motor cortex (M1; A, B) and ventral premotor cortex (PMv; C, D) of the intact monkey, and in the contralesional M1 (co-M1; E, F) and PMv (co-PMv; G, H) of the lesioned monkey (early stage). II–VI, layers II–VI of the cerebral cortex. I, J: Double-labeling study with SMI 32, an antibody against non-phosphorylated neurofilament H. I: Localization of SPP1 mRNA–positive neurons in layer V of the lesioned monkey (late stage). J: SMI 32 immunoreactivity in the same section as (I). Arrows indicate SPP1 mRNA–positive neurons showing SMI 32 immunoreactivity. Double arrowheads indicate SMI 32-immunoreactive neurons with no SPP1 expression. K: A bar chart showing the average density of SPP1 mRNA–positive neurons in M1, PMv, and dorsal premotor cortex (PMd) of an intact monkey, and in the co-M1, co-PMv, and contralesional PMd (co-PMd) of the lesioned monkey, with standard error. *P<0.05, **P<0.01, according to the Mann-Whitney U test. L: Scattergram showing the relationship between food retrieval success rate with precision grip and the density of SPP1 mRNA–positive neurons in the co-PMv. **P<0.01, according to linear regression analysis. The dashed and solid lines are linear approximations of the data represented by triangles and circles, respectively. The triangles and circles are data points from each lesioned monkey at early and late recovery stages, respectively. Scale bars  = 200 µm in A–H; 50 µm in I, J.

In intact monkeys, SPP1 mRNA hybridization signals were more prominent in layer V of M1 than in PMv (Fig. 6A–D), as described in our previous report [11]. After the l-CST lesion, in contrast, the signals were much less prominent in the contralesional (co-) M1 (co-M1; Fig. 6E, F), whose descending axons were primarily damaged by the lesion, than in the co-PMv (Fig. 6G, H). The expression area in the co-PMv included the posterior part of the inferior postarcuate bank, which contains the CST to the upper cervical spinal segments and the projections to the brainstem, neither of which were directly affected by the lesion [31], [52]–[54]. To investigate whether SPP1 was expressed in these subcortically projecting neurons during the recovery stage, we performed the following analyses: (1) double-labeling immunofluorescence with SMI 32 (intact monkey, n = 1; monkeys at early [n = 1] and late [n = 1] recovery stages), and (2) comparison of the average size of the Nissl-stained neurons to that of the largest cell bodies of the SPP1 mRNA–positive neurons in the co-PMv. Nearly all SPP1 mRNA–positive neurons in layer V of the co-PMv were labeled by SMI 32 in both the early and late recovery stage monkeys (arrows in Fig. 6I, J) but not vice versa (double arrowheads in Fig. 6I, J). The average size of the largest Nissl-stained neurons (see Materials and Methods) was nearly the same as that of SPP1-positive neurons in the co-PMv (Nissl, 569.63±37.46.78 µm^2^; SPP1, 509.53±38.72 µm^2^; mean ± standard error [SE]; n = 31 neurons [6 lesioned monkeys], *P* = 0.28, Mann-Whitney U test). These results indicate that SPP1 mRNA in the co-PMv is expressed in a subset of the neurons with the largest cell bodies. The majority of these neurons may be CST neurons that project to the upper cervical spinal segments, given that the cell bodies of CST neurons are larger than those of other projection neurons in layer V, including corticothalamic and corticostriatal neurons [48].

Quantitative comparisons between the areas of each conditioned monkey (intact monkeys, n = 3; lesioned monkeys, n = 9) confirmed that the density of SPP1 mRNA–positive neurons significantly decreased in the co-M1, but increased in the co-PMv after the l-CST lesion (Fig. 6K). To assess whether the level of SPP1 mRNA expression was related to the extent of functional recovery, we investigated the correlation between the density of SPP1 mRNA–positive neurons in the co-PMv and the success rate of food retrieval with a precision grip. A significant positive correlation (*P*<0.01) and a trend towards a positive correlation (*P* = 0.18) were observed at the early and late recovery stages, respectively (Fig. 6L).

## Discussion

Previously we suggested that SPP1 expression in M1 may reflect the functional or structural specialization of highly developed corticospinal systems in certain primate species [11]. This hypothesis is now further supported by the results of three different analyses: species differences, postnatal development, and functional/structural changes after a lesion of the CST at the mid-cervical level.

### Species differences

Although SPP1 was commonly expressed in the red nucleus and spinal cord of all species examined (Table 4; Fig. 3C–E), it was preferentially expressed in large neurons in M1 of species with highly developed corticospinal systems. However, the results observed in layer III of the capuchin monkey and human M1 indicate that SPP1 expression is not always correlated with neuron size, similar to our previous study [11]. Thus, the intense expression in large layer V neurons is likely related to long-descending projections, including CST [47], [48]. Of particular note are the differences in both the development of corticospinal systems and M1 SPP1 mRNA expression among the capuchin monkeys, squirrel monkeys, and marmosets, given that they are closely related species of New World monkeys. The density of SPP1-positive neurons in layer V of the squirrel monkey was much smaller than that in the capuchin monkey and larger than that in the marmoset. Unlike the marmoset, the squirrel monkey has a pseudo-opposable thumb that can be opposed to the side of the index finger; therefore, the squirrel monkey can perform tasks requiring the skilled use of digits, such as retrieving food pellets from small cylindrical wells [25], [55], [56]. The CST neurons of squirrel monkeys are more anatomically developed than those of marmosets and less developed than those of capuchin monkeys: cortico-motoneuronal projections are abundance in capuchin monkeys, sparse in squirrel monkeys, and absent in marmosets [14], [57], which are supposed to underlie the difference in finger dexterity. Thus, the differential M1 SPP1 expression levels among capuchin monkeys, squirrel monkeys, and marmosets may be related to the differential degrees of CST development.

We sometimes observed SPP1-positive neurons with relatively weak hybridization signals outside layer V in the cerebral cortex. These observations indicate that SPP1 plays a role in several populations of cortical neurons other than CST neurons, as described in our previous report [11]. In particular, a number of SPP1-positive neurons were observed not only in layer V, but also in layer III of the capuchin monkey and human M1 (Fig. 3A, B, E). Although the role of SPP1 in layer III is unclear, its expression may also be related to the functional/structural specialization of highly developed corticospinal systems that are responsible for finger dexterity because both the capuchin monkey and human use their fingertips to grasp small objects much more frequently than do other primates, including rhesus macaque [58]. Intrinsic connections within M1, arising primarily from layer III neurons, have been suggested to modulate the activity of motor cortical areas whose predominant output pathway is CST to different forelimb muscle groups that act in coordination [28], [59], [60]. Therefore, SPP1-mediated functional/structural specialization of intrinsic cortical circuits among different cortical modules, such as the increase in conduction velocity (see below for details), may be associated with the specialization of CST.

Previous studies have reported specific expression of particular molecules in some primate species. For example, the occ1/follistatin-related protein is expressed in primary visual cortex neurons of the rhesus macaque and marmoset but not in that of the mouse [61]. Similarly, paraneoplastic antigen-like 5 is expressed in association cortex neurons of the rhesus macaque and marmoset but not in that of the mouse [62]. Compared to the expression of these genes, SPP1 expression in M1 is unique in that it is different among primates and even among New World monkeys (i.e., marmoset, squirrel monkey, and capuchin monkey).

The present results demonstrate the importance of comparative gene expression studies in primates. Humans execute highly sophisticated brain functions despite not having an exceptionally large number of protein-coding genes among mammals [63]. One of the causal factors behind such sophisticated functions may be specific gene expression in particular brain areas. Some non-human primate species can perform similar functions to those that are particularly developed in humans, such as finger dexterity, tool use [64], and vocal communication [65], and are believed to share common neural mechanisms. Thus, a comparative gene-expression analysis between closely related primate species with and without such functions will allow us to understand the molecular bases of human brain function.

### Postnatal development

The density of SPP1 mRNA–positive neurons in the macaque M1 increased logarithmically with age after birth. Previous studies have reported a developmental change in SPP1 expression in the rat brainstem and cerebellum [66] and in the mouse spinal cord [67]. These reports showed that SPP1 expression was greater in these areas during the late embryonic stage and reached an adult expression level in the early postnatal stage (i.e., P14 [2 weeks]). In contrast, macaque monkey M1 began to express SPP1 after birth and required P957.6 days (2.6 y) to attain the adult expression level. Olivier et al. (1997) reported that the conduction velocity of the macaque monkey CST increased logarithmically up to 36 months and suggested that these changes may be compatible with the size and degree of CST myelination. Moreover, our previous studies reported that SPP1 is preferentially expressed in neurons with high conduction velocities [11], [40], and another study suggested that SPP1 regulates the axonal myelination process [68]. Taken together with these previous reports, the present results suggest that SPP1 may be involved in the myelination of CST axons during postnatal development, which underlies the increase in conduction velocity.

### Changes after lesion of l-CST

The present results showed that better recovery of finger dexterity was associated with higher SPP1 mRNA expression in the co-PMv after the l-CST lesion at the mid-cervical level. This result is consistent with our previous studies, which suggest functional and structural changes in the co-PMv after an l-CST lesion [19], [69]. The CST to the upper cervical spinal segments, which was not directly affected by the lesion of the mid-cervical level, arises from the PMv. Therefore, this projection has been suggested to functionally compensate for the lesioned l-CST [69]–[73]. Although further study is needed to clarify the role of SPP1 during the recovery of finger dexterity, we speculate that increased expression of SPP1 in the co-PMv may be involved in the increased conduction velocity of the CST from the PMv, which has the potential to control hand movements via propriospinal neurons in the upper cervical segments [31], [52], [53], [74].

The present study revealed the expression of SPP1 in the central nervous system of the several different species, during postnatal development, and after l-CST lesion. Our results from all three analyses suggest that SPP1 expression influences the functional or structural specialization of certain corticospinal systems. Although the function of SPP1 in neurons remains to be elucidated, our results suggest that SPP1 expression may be involved in the development of a certain neuronal population with high conduction velocity through the process of axon myelination. To obtain more direct evidence regarding the role of SPP1, virus-induced regulation of primate gene expression is necessary [75]. The development of this technique is an important challenge for future brain research because a list of genes whose expression is specific to certain primates has been accumulated, as described above.
